# Consumer-grade EEG devices: are they usable for control tasks?

**DOI:** 10.7717/peerj.1746

**Published:** 2016-03-22

**Authors:** Rytis Maskeliunas, Robertas Damasevicius, Ignas Martisius, Mindaugas Vasiljevas

**Affiliations:** 1 Multimedia Engineering Department, Kaunas University of Technology, Kaunas, Lithuania; 2 Software Engineering Department, Kaunas University of Technology, Kaunas, Lithuania; 3 Computer Science Department, Kaunas University of Technology, Kaunas, Lithuania

**Keywords:** BCI, Consumer-grade EEG, Usability

## Abstract

We present the evaluation of two well-known, low-cost consumer-grade EEG devices: the Emotiv EPOC and the Neurosky MindWave. Problems with using the consumer-grade EEG devices (BCI illiteracy, poor technical characteristics, and adverse EEG artefacts) are discussed. The experimental evaluation of the devices, performed with 10 subjects asked to perform concentration/relaxation and blinking recognition tasks, is given. The results of statistical analysis show that both devices exhibit high variability and non-normality of attention and meditation data, which makes each of them difficult to use as an input to control tasks. BCI illiteracy may be a significant problem, as well as setting up of the proper environment of the experiment. The results of blinking recognition show that using the Neurosky device means recognition accuracy is less than 50%, while the Emotiv device has achieved a recognition accuracy of more than 75%; for tasks that require concentration and relaxation of subjects, the Emotiv EPOC device has performed better (as measured by the recognition accuracy) by ∼9%. Therefore, the Emotiv EPOC device may be more suitable for control tasks using the attention/meditation level or eye blinking than the Neurosky MindWave device.

## Introduction

### Motivation

Human–computer interaction (HCI) devices allow humans to interface with computers for the purposes of data entry, control or communication. Most of the efforts over the years have been dedicated to the design of user-friendly, efficient and ergonomic systems to produce a faster and more comfortable means of communication. Natural User Interfaces (NUI) (Jeong et al., 2004) based on voice recognition, gesture recognition, physical movement and other technologies have received enormous research attention over the years and successful examples of these technologies are being produced commercially.

Recently, a radical and novel approach of computer interfaces has received a lot of scientific interest. Since the first experiments of electroencephalography on humans in 1929, the electroencephalogram (EEG) of the human brain has been used mainly to evaluate neurological disorders in the clinical environment and to investigate functions of the brain in the research laboratory environment. An idea that brain activity could be used as a communication channel has gradually emerged. An electroencephalogram (EEG) demonstrates direct correlations with user intentions (Dong & Lee, 2012), thereby enabling a direct Brain-Computer Interface (BCI) communication.

BCI is a communication channel that enables users to control devices and applications without the use of muscles. BCI research has been successfully used not only for helping the disabled, but also as being an additional data input channel for healthy people to be used as an extra channel in game control, augmented reality applications, household device control, fatigue and stress monitoring and other applications. BCI design represents a new frontier in science and technology that requires multidisciplinary skills from fields such as neuroscience, engineering, computer science, psychology and clinical rehabilitation. The ultimate goal of BCI research is to create a system that responds to users’ modulation of his/her brain signals (Hammon & De Sa, 2007) and gives feedback to the user.

Despite recent developments, there are numerous obstacles to building a usable and effective BCI system. Implementing BCI requires high computational capacity to analyze brain signals in detail and in real-time, and such equipment was very expensive. Current consumer-grade BCI systems are inaccurate and have a low transfer rate. This means that the user may need a long period of time in order to send commands to the device that is being controlled.

Multi-electrode, medical grade EEG systems have long been used in hospitals and laboratories. But the recent availability of inexpensive, single-channel, dry-electrode EEG devices makes it feasible to take this technology outside of the laboratory into informal real-world environments such as schools and homes. The benefits of such devices are affordability and ease of use. Considering the current availability of many different commercial consumer-grade EEG devices, there is a need in exploring the feasibility of using low-cost EEG devices for monitoring individuals’ EEG signals in their natural environment. While a system with a larger number of electrode/sensors would be providing more and better quality data than its counterpart, the users of the domestic applications of the BCI technologies such as neurofeedback games (Thomas, Vinod & Guan, 2013; Yisi, Sourina & Hou, 2014) favor lightweight easy-to-use EEG headset with a small number of sensors. Therefore, evaluating the applicability and feasibility such devices for non-medical applications is important.

The aim of this article is to analyze the suitability of using consumer-grade EEG devices for simple control tasks using the attention/meditation levels and blinking recognition. Here control task is understood as manipulation of an external object (physical or virtual) using EEG of a subject as an input. The importance of research is motivated by an increased number of low cost customer-grade EEG devices appearing both on the market as well as increasingly cited in the BCI and EEG research domain papers.

### Attention and meditation states

There has been a great deal of research works focusing on detecting the attention and relaxation (meditation) states of mind from the characteristics of EEG (Aftanas & Golocheikine, 2001; Jacobs & Friedman, 2004; Lin & John, 2006; Hamadicharef et al., 2009; Rebolledo-Mendez et al., 2009; Jiang et al., 2011; Li et al., 2011; Liu, Chiang & Chu, 2013; Patsis et al., 2013; Vyšata et al., 2014; Kaur & Singh, 2015). The detection of the attention and meditation is important in many fields, including clinical studies of stress reduction, sleep deprivation, fatigue, educational studies of learner attention and game studies of player concentration and engagement.

Relaxation is an integrated body reaction that reflects the voluntary resting state of both the body and the mind (Teplan, Krakovská & Špajdel, 2014); however, it is difficult to define in terms of specific physiological parameters. According to Travis (2001), five categories of physiological variables have the potential to discern between the levels of relaxation, including breath and heart rates, as well as skin conductance. Similar parameters for the characterization of stress levels were reported by Vavrinský (2005). However, there is no consistent evidence regarding the characterization of relaxation in terms of the specific EEG features. The increased power and acute changes of the alpha and theta activity (Jacobs & Friedman, 2004) and frontal alpha coherence (Travis, 2001), are usually considered to be neurophysiological indicators of a state of rest. Alpha band is predominant in a relaxed adult, while theta band is prominent in light sleep. The ratio of signal power in alpha and theta bands is also used to assess relaxation. One research indicates that the sum of alpha and theta, and the sum of alpha, beta and theta are good indices for measurement of neurological relaxation (Lin & John, 2006).

Relaxation can be difficult to reach in real-world environment by modern people due to an increased tempo of life, stress, surrounding noise, etc. Various types of psychosomatic techniques such as meditation are known to induce a relaxed state (Aftanas & Golocheikine, 2001). But neuroelectrophysiology of the meditation-induced mental states is still an open question (Kaur & Singh, 2015). Vyšata et al. (2014) verified the existence of statistically significant differences between certain features of EEG data (such as reduction in permutation entropy) before meditation and during meditation.

Attention, on the other hand, has been defined as the ability to focus our cognitive resources on one relevant aspect of the environment while ignoring other irrelevant aspects (Riccio et al., 2013). Many BCI-based neurofeedback games (Wang, Sourina & Nguyen, 2010; Jiang et al., 2011; Pires et al., 2011) employ attention-related EEG feature as the control parameter, as attention is a key factor of human cognition. However, automatic determination of subjects’ attention state is challenging because attention involves complex human cognitive functions. Previous research (Liang et al., 2005; Hamadicharef et al., 2009; Li et al., 2011) has demonstrated evidence that EEG signals (esp. the beta band) contain considerable information about attention, indicating the possibility of recognizing a subjects’ attention level by studying the EEG data.

### Consumer-grade EEG devices

Several types of low-cost EEG devices exist commercially in the market today. Further, we consider two consumer-grade EEG systems: Emotiv EPOC (https://emotiv.com/epoc.php) and Neurosky MindWave (http://neurosky.com/)

**Figure 1:**
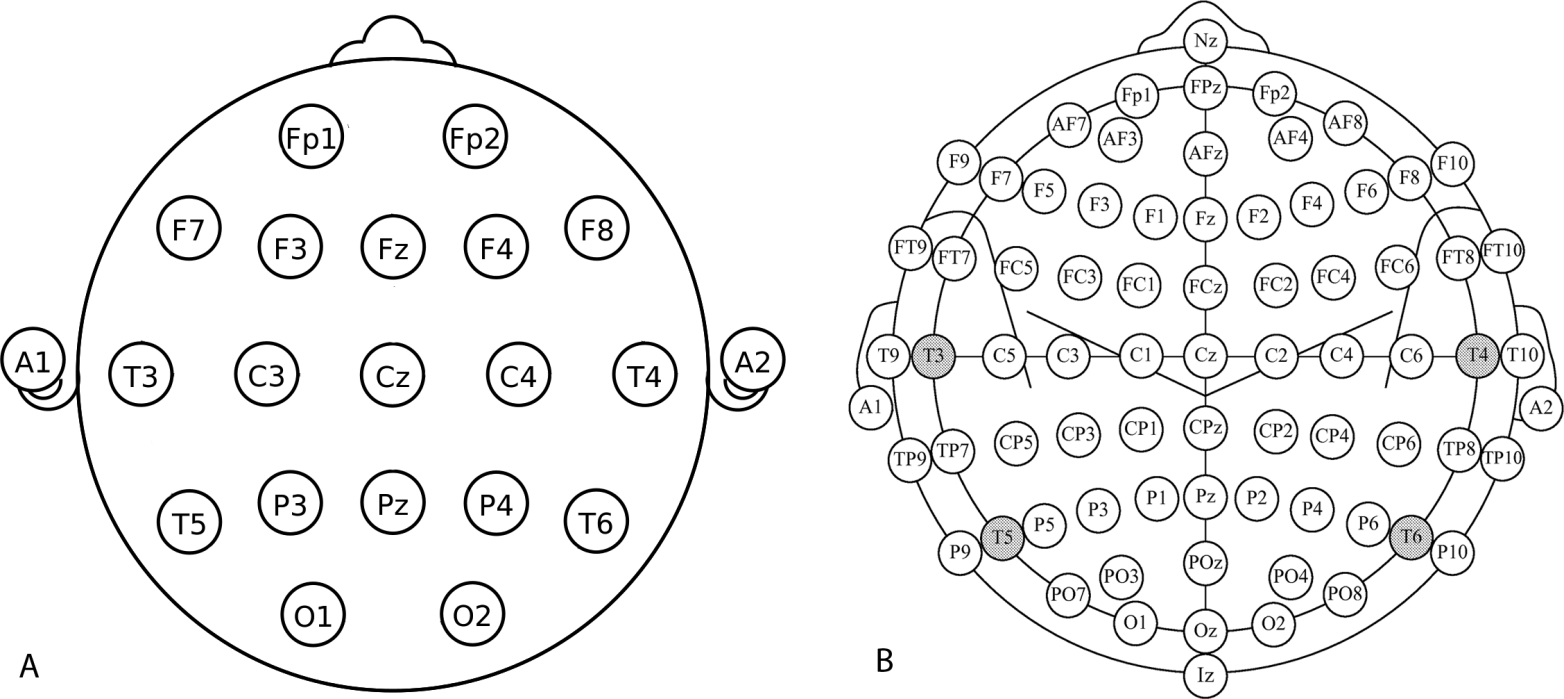
Electrode placement according to the International 10–20 (A) and 10–10 (B) system. Odd electrode numbers are on the left hemisphere, even electrode numbers on the right hemisphere. Letters correspond to lobes–F(rontal), T(emporal), P(arietal), and O(ccipital). C stands for Central (there is no central lobe).

According to Stamps & Hamam (2010), the most usable low-cost EEG device is the Emotiv EPOC headset. The Emotiv EPOC is a lightweight inexpensive EEG device. The EPOC was not originally intended for research; however, it is becoming increasingly popular due its flexibility and wide range of suites which it offers. The EPOC device comes with a series of software suites that can detect user’s emotions, facial expressions and control objects in a virtual world. The Emotiv EPOC device has 14 electrodes and two reference electrodes, placed in the 10–10 international system of EEG electrode placement (see Fig. 1) (Jurcak, Tsuzuki & Dan, 2007). The headset is designed as a video game accessory where developers are interested in using the device as a controller. We used the Research Edition, which provides both the interface for programming with the headset and access to raw EEG data. The internal sampling rate of the device is 2048 Hz. The data is then downsampled to 128 Hz before becoming available to the system for capturing the EEG signals. The captured data contains values for each of the 14 electrodes on the EPOC headset. Kos’myna & Tarpin-Bernard (2013) examined multimodal combinations of BCI and eye tracking in the context of a simple puzzle game involving tile selection and rotations using Emotiv EPOC. The results of the experiment performed on 30 subjects show that when using BCIs, Steady State Visually Evoked Potentials (SSVEP) manages to lead to a performance using Linear discriminant analysis (LDA) classifier (the average classification accuracy for SSVEP was 79.8% with a standard deviation of 1.3%) very close to pure eye tracking, which is commendable but still is perceived as a not too natural way of performing a rotation. As for the Motor Imagery, the performance (61.3% for the classification accuracy with a standard deviation of 4.3%) greatly suffered from the low classification accuracy that led to many errors and much slower interactions. Azcarraga & Suarez (2001) suggested that the Emotiv EPOC needs a small amount of time to normalize a user’s data. In their study they told the subjects to relax for a period of three minutes in order for the EEG to allow to create a baseline for EEG data. Duvinage et al. (2013) have demonstrated that the performance of EPOC is above random and that EPOC could be used for non-critical uses such as games for healthy people or communication for disabled users. In Liu et al. (2012), a g.tec device was compared with EPOC showing worse (around 10%) results for the EPOC. Chumerin et al. (2013) used Emotiv for a game control in a real-world setting achieving mean accuracy of 80.37%. Lin, Wang & Jung (2014) used Emotiv to perform online SSVEP decoding in human walking. Haapalainen et al. (2010) achieved an average classification accuracy of 60.2% across the various cognitive experiments they conducted using data from various bio-sensors, including the attention signal. Despite low accuracy, the authors claim the consumer-level EEG devices can be deployable to users in natural environments and in real-world BCI systems.

According to Stamps & Hamam (2010), Neurosky MindWave is at the lower end of usability of low-cost consumer EEG devices. It has only one electrode (placed at Fp1) and a reference electrode near the ear, placed in the 10–20 system of EEG electrode placement. As with other devices it can also detect blinking. The manufacturers of Neurosky MindWave claim that above the typical EEG device functionality it is also capable of detecting two mind states (focused and are relaxed). The signal is processed 128 times per second. In order to utilize a device for research purposes, a researcher must program additional functionality via provided SDK. Patsis et al. (2013) used Neurosky MindWave to measure the attention levels of Tetris players with respect to game difficulty. The results show that the trend of attention value rather than actual values can be related to game difficulty, that non-linear increase in game difficulty results in a non-linear increase in attention. Crowley et al. (2010) evaluated the NeuroSky Mindset headset for measuring the attention and meditation levels of a subject while conducting two psychological stress-inducing tests (Stroop’s and Tower of Hanoi) and have achieved 78% accuracy. However, they were unable to correlate isolated moments of human error in Stroop’s test (Stroop, 1935) with a precise change in the attention or meditation signals.

Vourvopoulos & Liarokapis (2014) tested both the Neurosky headset and the Emotiv headset. Evaluation results indicate that robot navigation through commercial BCIs can be effective and natural both in the real and the virtual environment. Shirazi et al. (2014) have achieved recognition of reading vs. other tasks with 74.4% and relax vs. others with 79% on average. However, there was significant variability in the recognition results since 6 participants out of 15 had reading recognition rate less than 50%.

Badcock et al. (2015) assessed the validity of the Emotiv EPOC gaming EEG system as an auditory event-related potential (ERP) measurement tool in children. The results were validated by simultaneous measurement using a research-grade Neuroscan system and the EPOC system. The study has obtained intra-class correlation of 0.67–0.74 between mismatch negativity ERP components recorded several problems with using the EPOC device such as the need of the shielded room to reduce EEG noise, a smaller number of recorded acceptable epochs, instability of EPOC’s sensors resting on the scalp, and delayed latencies for the EPOC system.

For recording the state of eyes (open or closed) Roesler et al. (2014) have compared Neurosky MindWave and Emotiv EPOC devices. The results show that the MindWave headset is not suitable for the classification of the eye state (classification accuracy only 43.52%) therefore it is not useful for the control tasks. However, the EPOC headset has shown high performance for eye state prediction (mean error rate only 10.5%).

### BCI illiteracy

While BCI theoretically can be employed with impaired subjects as well as with healthy subjects, there are problems that must be resolved before BCI can be adopted to the wide use by the public. It has been noted the success in using BCI systems significantly depends upon individual characteristics of its users. In existing BCI systems, 20–30% of users are known to show significantly worse performance than others (Dickhaus et al., 2009). This causes high performance variability both between and within subjects. The phenomenon is known as BCI illiteracy (Ahn et al., 2013a; Ahn et al., 2013b). This phenomenon has been underresearched, and a clear understanding of the BCI illiteracy or a solution to this problem has not been reported so far. In a study done by Popescu, Blankertz & Müller (2008) with a total of 23 untrained users. About 20% of subjects did not show strong enough motor-related mu-rhythm variations for effective asynchronous motor-imagery BCI, another 30% exhibit slow performance (<20 bits/min). Another study (Badcock et al., 2015) claims to have achieved better results of using BCI with children than with adult people leading to speculation of whether aging related effects have influence over ability to use BCI effectively. The boundary between BCI literate and BCI illiterate also has not been defined conclusively. Several studies (Kubler & Muller, 2007; Allison et al., 2010) defined 70% accuracy as the threshold value. However, the number is too close to the average performance of subjects in using BCI. However, there has not been enough research done to prove that this distinction is meaningful and other thresholds could substantially affect results. Therefore, the problem of BCI-illiteracy needs to be understood so that BCI systems could be useful in the future.

There is still no clear cause as to why some of healthy BCI users exhibit “illiteracy” with BCI systems by being no able to generate controllable brain signals, and how it could be overcome using the computational intelligence methods. All healthy brains have the same structures in roughly the same places, however some cortical areas may not produce EEG signal detectable on the scalp. For example, this may result from the folding of the brain (Fialek, 2014). The performance of BCI may also be affected by other psychological and cognitive factors such as stress, mood, fear of BCI technology, insufficient instructions, unsuitability of control tasks or the flaws of BCI training protocols. Understanding this phenomenon better and investigation of its roots are important in improving usability of BCI systems. Predictors of BCI performance may help to avoid the frustrating and costly procedure of trying to establish control over BCI, while also help in early illiteracy detection (Sannelli et al., 2008). Motor imagery (MI) questionnaires can help detecting BCI illiterate so they can be trained to overcome BCI illiteracy prior to using BCI (Vuckovic, 2010) by using specific neurofeedback training procedures as described by Vidaurre & Blankertz (2010). Popular MI questionnaires are Hall’s questionnaire (Hall & Pongrac, 1983), Movement Imagery Questionnaire-3 (MIQ-3) (Hall & Martin, 1997) or Kinaesthetic and Visual Imagery Questionnaire (KVIQ) (Malouin et al., 2007). Any subject with this phenomenon would be excluded from further analysis of data obtained from BCI.

### Technical aspects of EEG recording

Each change in the EEG recording, not directly influenced by the human brain electrical potential, is called an artifact. Noise sources that cause this artificial activity can be classified into sources of biological and sources of technical origin. Biological artefacts have their roots in the human physiology. They are mostly voltage differences on the scalp caused by extra-cerebral voltage sources, such as muscles. Technical artefacts arise from electrical sources in the pathway from the electrodes to the EEG recording device. They can also be caused by external electromagnetic activity or static electric fields. The recognition and suppression of artefacts is an important problem in electroencephalography. The origin and characteristics of different artefacts is also important in recognizing and minimizing their influence. Some artefacts can be similar to real EEG activity. Artefacts, just like EEG features, have to be diagnosed and interpreted. While technical artefacts increase error rates in BCI systems, biological artefacts can cause artificial effects, i.e., eye movement can be a major source of noise in EEG. If different eye movements are registered in two different experiments, it is hard to recognize, if EEG activity was also different.

EEG potential differences are in the range of 10–100 µV, and therefore have to be registered by sensitive amplifiers. It is obvious that EEG recordings are full of outside electrical interferences—artefacts. Mostly these artefacts differ significantly from the human brain activity and can be easily eliminated. Yet, in some cases they are very similar or overlap brain activity. Artefacts are removed by sophisticated modern equipment, good scalp electrode placement, optimization of the electrical current in the electrodes, filters for known artifact sources, elimination of large artefacts, exceeding 1 mV and EEG variations exceeding 200 µV. This allows for the control of artefacts or their complete removal. Below we discuss a list of the most common EEG artefacts (Fisch & Spehlmann, 1999):

1. Lid and eye artefacts are the most common extra-cerebral noises in EEG recording. In slow cortical potentials (SCP) recording, these types of artefacts can be difficult to control. The easiest way to reduce eye interference is gaze fixation: the subject is asked not to blink during the duration of the test. Most subjects find gaze fixation tiring and avoid or ignore it. It is better to measure eye artefacts with an electrooculogram (EOG). This is performed by placing electrodes next or onto the person’s eyebrows and excluding samples with high potential changes in these electrodes altogether.

2. Pulse artefacts are noise on single electrodes. They arise when an electrode is placed on a pulsating blood vessel. It is a problem of dry, close positioned or defected electrodes. These artefacts are easily differentiated from EEG—the pulse has a uniform pattern.

3. ECG artefacts are influencing reference point of measurements. The ECG is a consequence of a strong muscular dipole, caused by the human heart, which reaches out through the whole human body. It can be 10 times the magnitude of EEG brain waves. ECG patterns can be removed digitally after recording.

4. Muscle artefacts recorded in the EEG. Therefore these artefacts are recorded over the temporal and frontal scalp areas, where face and neck muscle movements are the main cause of interference. These artefacts can be removed by low-pass filters. Grand averaging also helps in removal, because muscle artefacts are random in time and amplitude.

5. Movement artefacts arise from subject’s body or head movements. The electrode is thereby mechanically moved by the connecting cable. All artefacts arising from muscle movement (i.e., face mimics), breathing and jaw movement artefacts are regarded to as movement artefacts. Movement artefacts, therefore, are recorded together, mixed in with the muscle artefacts.

6. Skin and sweat artefacts introduced by changes in skin potential (Lutzenberger et al., 1985). They appear as low frequency waves (0.2–1 Hz) in the EEG, are irregular and can be correlated to a presented stimulus. They can be detected by a characteristic form of negativity of 2–3.5 s, followed by a positive drop. These obstacles cause adverse effects in signal measurements with high leakage currents and high contact impedances leading to corruption of EEG signals and the quality of measured signals becoming lower than desirable (Lee et al., 2013). Some cosmetic chemicals and hairsprays can also influence electrode potentials. Sweat artefacts are normally recognized by big, synchronous potential changes in several recording channels, mostly in the forehead region. They are partially influenced by laboratory temperature control.

Technical artefacts that influence the quality of the EEG signal are as follows.

1. Electrode and cable artefacts. Reference electrode movement is categorized under biological sources, because it is caused by the movement of the subject (unrest, cough etc.). Electrode artefacts can lead to positive or negative potential changes, but they do not follow any specific rules. Their appearance, however, depends on the type of measurement mode being used. With a reference point measurement, the noise in the reference leads to a noise in all recording electrodes. These artefacts are mostly distinguished by sharp potential changes and are easy to detect. Electrode short circuits, however, are difficult to notice. They are caused by conductive fluid pathways between neighboring electrodes: strong sweating or an excess of conductive gel. Electrode artefacts can also arise from cable connector faults, caused e.g. by prolonged exposure of the electrode in salt solutions, which causes a chlorination effect on the contact and can lead to false voltages. A light soiling in the contacts can influence resistance. After a period of use, cable cracking can occur.

2. The 50 Hz power line frequency and other electromagnetic interferences. Electromagnetic interferences are artefacts that are caused by alternating current (AC) devices in the vicinity of very sensitive EEG measurement devices. These primarily arise from the 50 Hz (in European countries) alternating current sources such as light and device supply nets. Another problem involves a bad grounding of the subject. A capacitive effect is caused by electrical currents in the laboratory wall and other devices near the subject. Typical sources include radio and microwaves. These do not appear in low frequency EEG, but are converted to a small low frequency voltage in the amplifiers, in a process of demodulation.

3. Electrostatic interference is caused by rubbing static electricity, causing large electrical potentials on the subject. These can lead to artefacts in EEG, when a ground current between a subject and recording equipment arises.

## Methods

Each individual person is very different. Each of us thinks differently. Each of us has different brain “signal” strength. Due to this individuality every user must have his/her own individual profile best describing his physical “signal generating” capabilities. These capabilities of course are still variable and not completely universal (one can produce different signal on different days, physical status, mood, stress, sleep time, etc.). Ten (10) people participated in our experiment (nine males (subjects no. 1–9), one female (subject no. 10), aged from 24 to 31). All participants claimed to be physiologically and psychologically healthy.

We have used both EEG devices for the measurement and evaluation of attention and meditation levels, as well for blinking recognition. The methods used are detailed below.

### Recognition of attention and meditation states

We have tried to measure individual waveforms and produce some insights on the following conditions:

∙ A person is concentrating. As our test was a simple control task (just one command) we have asked to look at a picture(s) with a simple random calculus task and think of an answer. A task was of varying difficulty based on a time we wanted a participant to be concentrated (e.g., calculating linear equations in the form of 5*x*--10 = 0, 5*x*^2^ − 4*x* − 1 = 0, etc.). To verify that a person was really concentrated on calculating we have asked to say a result when they have it (no measurement was done during the “reveal of result” due to impacts on signal to working motorics, etc.)
∙ A person is relaxed. This data set was recorded when a person was not doing anything. In the case of control task - no command was produced at this stage (think as idle state).

The EEG device provides two metrics: attention and meditation. These two signals are derived from the EEG signal using proprietary techniques. The attention value reflects the intensity of a user’s level of mental “focus” or “attention” during increased mental effort, while the meditation value point to user’s mental “calmness” or “relaxation.” Each metric provides a relative measure of state from 0 to 100, these are not absolute values, where a value from 1 to 20 indicates “strongly lowered” levels of the state, and a value from 80 to 100 points to heightened levels of that state. Note that simply relaxing muscles may not result in increased meditation level as meditation is a person’s mental condition. Chi, Jung & Cauwenberghs (2010) noted that dry electrodes, such as the one in the Mindset, are limited to niche, nonmedical/scientific applications like toys, neurofeedback games and fitness monitoring. There are even fewer studies done using a single-channel EEG device. Most of the existing literature based on the Neurosky Mindwave was published just a few years ago, primarily on the proprietary attention and meditation signals.

**Figure 2:**
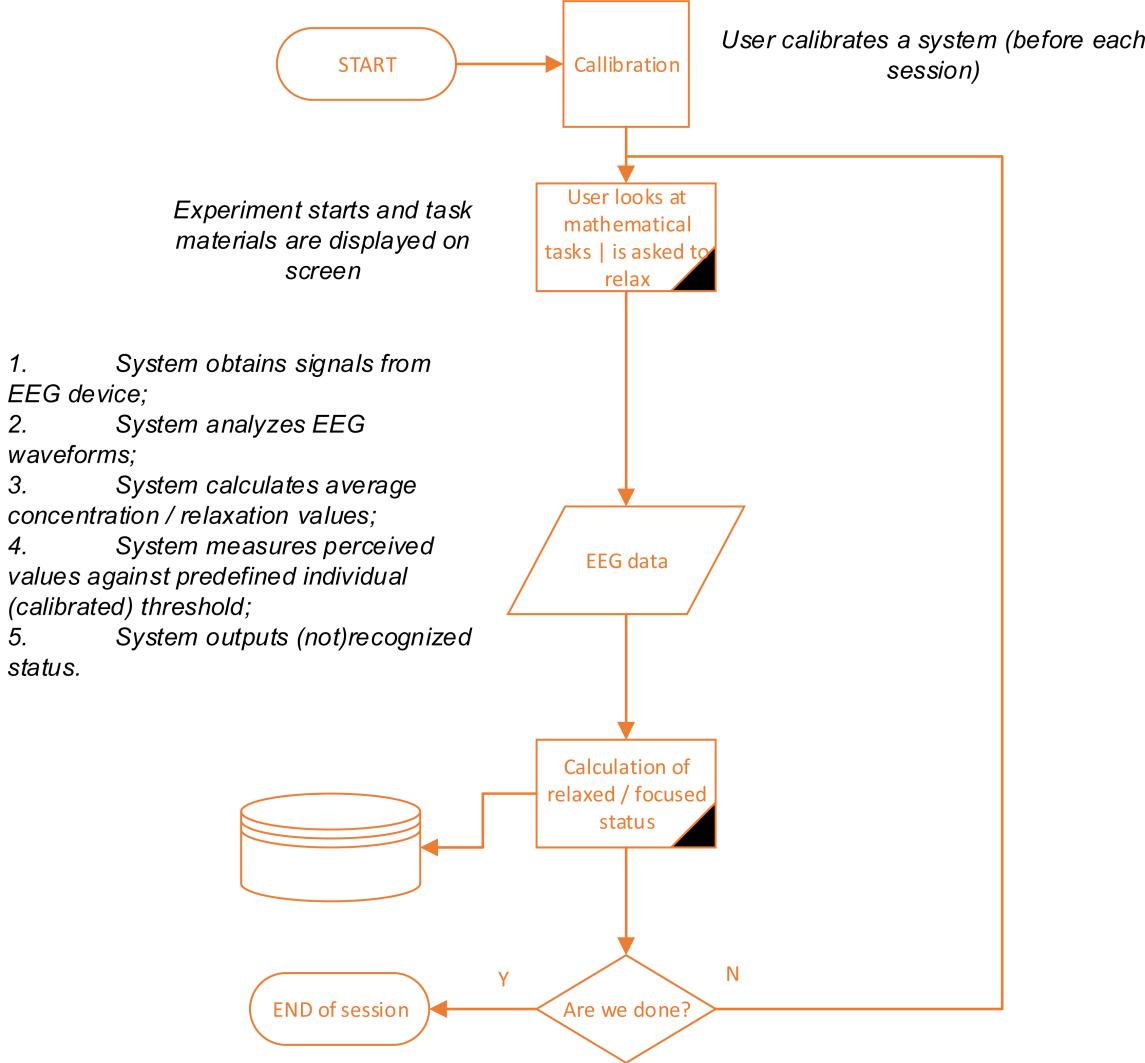
Organization of the experiment.

The organization of the experiment is detailed in Fig. 2. A perceived mental status (stressed/relaxed) of a subject is selected by computing the average intensity value (provided by standard functions in both manufacturers’ SDKs) and the standard deviation of the approximated EEG signal. Data has been indexed according to estimated values based on the profile condition (individually trained for each of the participants) as described below:

\begin{eqnarray*}{S}_{F}(u)= \left\{ \begin{array}{@{}ll@{}} \displaystyle 1,&\displaystyle \text{if}~T(u)\lt \mu \pm {\delta }_{u}\\ \displaystyle 0,&\displaystyle \text{otherwise} \end{array} \right. \end{eqnarray*}

\begin{eqnarray*}{S}_{R}(u)= \left\{ \begin{array}{@{}ll@{}} \displaystyle 1,&\displaystyle \text{if}~T(u)\gt \mu \pm {\delta }_{u}\\ \displaystyle 0,&\displaystyle \text{otherwise} \end{array} \right. \end{eqnarray*}

here: *S*_*F*_, focused; *S*_*R*_, relaxed; *μ*, trained threshold; *T*(*u*), measured approximated signal; *δ*_*u*_, standard deviation.

Subject profiles were made before each session, logging approximated values every 1 s, by asking each participant to concentrate on mathematical task or relax for 5 min before each of the experiments. Data gathered over the course of the experiment was compared with each value sets using the K-nearest neighbor (KNN) method.

### Blinking recognition

The consumer-grade EEG devices are often used for electromyography (EMG). The most often and easy to read and reproduce signal is an eye blink. Tested devices have a coded function for “strength” of a blink, but after a blink a numeric value is returned very second, but remains constant till a new blink (even a minor one) is detected. There was a problem for some participants that after a “strong and fast” blink some high value was reached but afterwards blinks were “weaker” reaching its limitations and not capturing (not function outputting as such) minor blinks. To overcome this, we have dropped a built in functionality and have made additional profiling for blinking, by asking each participant to blink for approximately 100 times (blink continuously for two minutes) and registering raw signal. Raw signal analysis allowed determining specific blinking patterns for each of the participants and avoiding fake signal cases such as head movement (often registered as a blink when using built-in functions). A measured blinking level was compared with an individual profile during the experiments. A gaze tracking device (EyeTribe) was used to monitor and verify if a person has blinked or not as it was faster than a human person and more accurate to detect fast and natural blinks of a user.

As there is no known methodology on how often a signal must be queried to get a reliable approximation we have chosen various signal fixation times. We have decided that to confirm one command 10 readings must be taken (from the initial concentrated mindset catch) at approximations calculating every 1, 2 and 5 s. We believe that longer time intervals are impractical for control tasks if one command takes longer than a minute to produce.

## Results

Our experimental results are presented for two research experiments. The first one considered the measurement and evaluation of attention and meditation levels, while the second experiment focused on blinking recognition.

### Recognition of attention and meditation states

**Figure 3:**
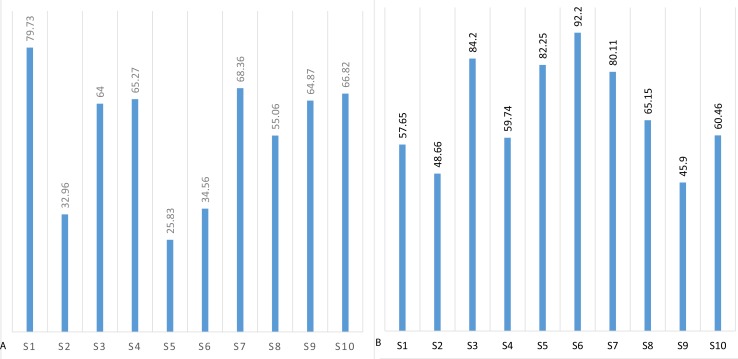
Mean threshold levels of attention obtained using Neurosky (A) and Emotiv EPOC (B).

**Figure 4:**
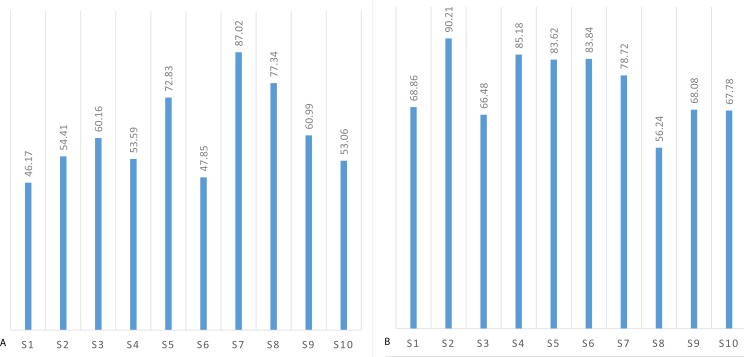
Mean threshold levels of meditation using Neurosky (A) and Emotiv EPOC (B).

The mean threshold attention and meditation recognition levels obtained using the methodology described in ‘Recognition of attention and meditation states’ (user profiling) with the Emotiv and Neurosky devices are presented in Figs. 3 and 4, respectively.

**Figure 5:**
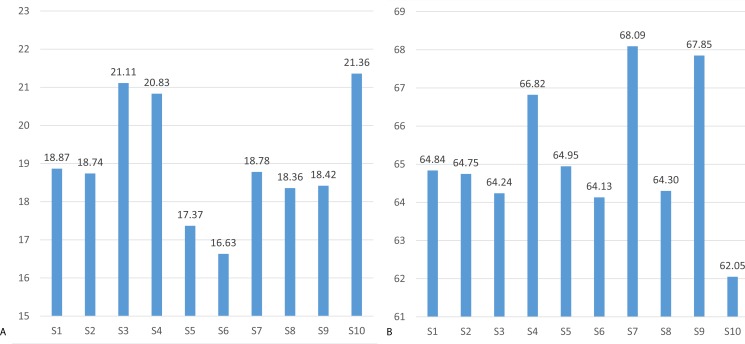
Mean recognition accuracy of attention state using Neurosky (A) and Emotiv EPOC (B).

**Figure 6:**
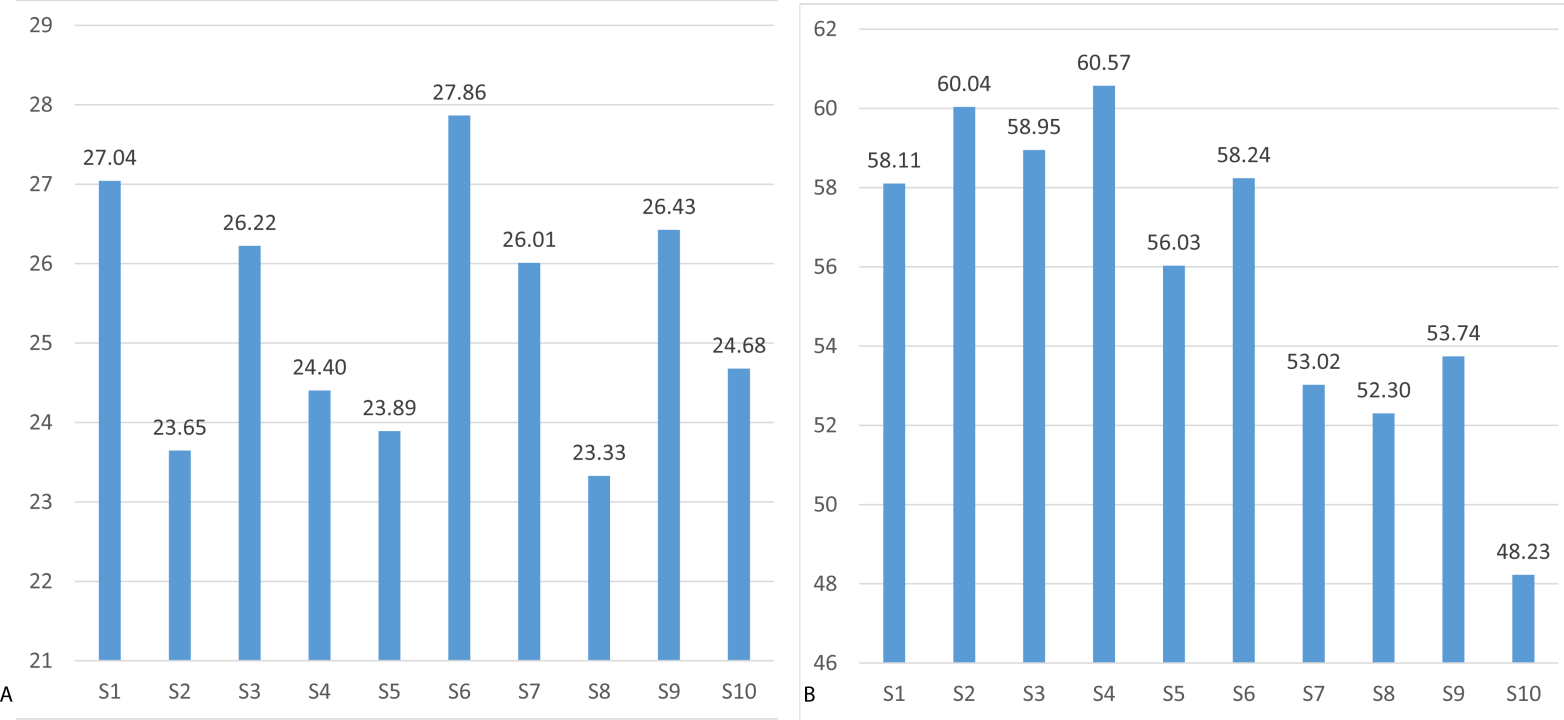
Mean recognition accuracy of meditation state using Neurosky (A) and Emotiv EPOC (B).

The mental state recognition accuracy (mean values) when performing concentration and relaxation tasks are summarized in Figs. 5 and 6, for attention and meditation states respectively.

The descriptive statistics of the Neurosky and Emotiv data for attention and meditation levels is presented in Table 1. Note high skewness results (>1) of attention recognition data using the Neurosky device. Positive skewness of 1.2865 indicates a piling of the values at the left side of the distribution for the Neurosky data, giving an indication of low values in the distribution. That result can be explained by low responsiveness from the users while using the device.

The results of a test of normal distribution showed that the data is not normally distributed (Shapiro–Wilk test (alpha = 0.95), mean *p* < alpha). The reasons for non-normality of the data may be extreme values in the data due to measurement errors, more than two processes generating data are overlapping (which could indicate strong noise signal added to the EEG signal), accumulation of round-off errors or the use of the measurement device with poor resolution, or that data follows a different distribution than normal. These results are contrary to the ones reported by Rebolledo-Mendez et al. (2009) for the NeuroSky MindBuilder device.

**Table 1:** Descriptive statistics of attention vs. meditation data.

Metric	Min	Max	Mean	Std.	Skewness	Shapiro–Wilk normality test	
						*W*	*p*
***Neurosky MindWave***							
Attention	0.05	74.79	19.05	12.95	1.2865	0.8780	0.1131
Meditation	0.31	49.79	25.35	17.75	−0.0150	0.9435	0.1405
***Emotiv EPOC***							
Attention	50.00	79.79	65.20	8.57	0.0028	0.8836	0.1878
Meditation	20.21	89.86	55.92	20.78	−0.0535	0.9394	0.1261

**Figure 7:**
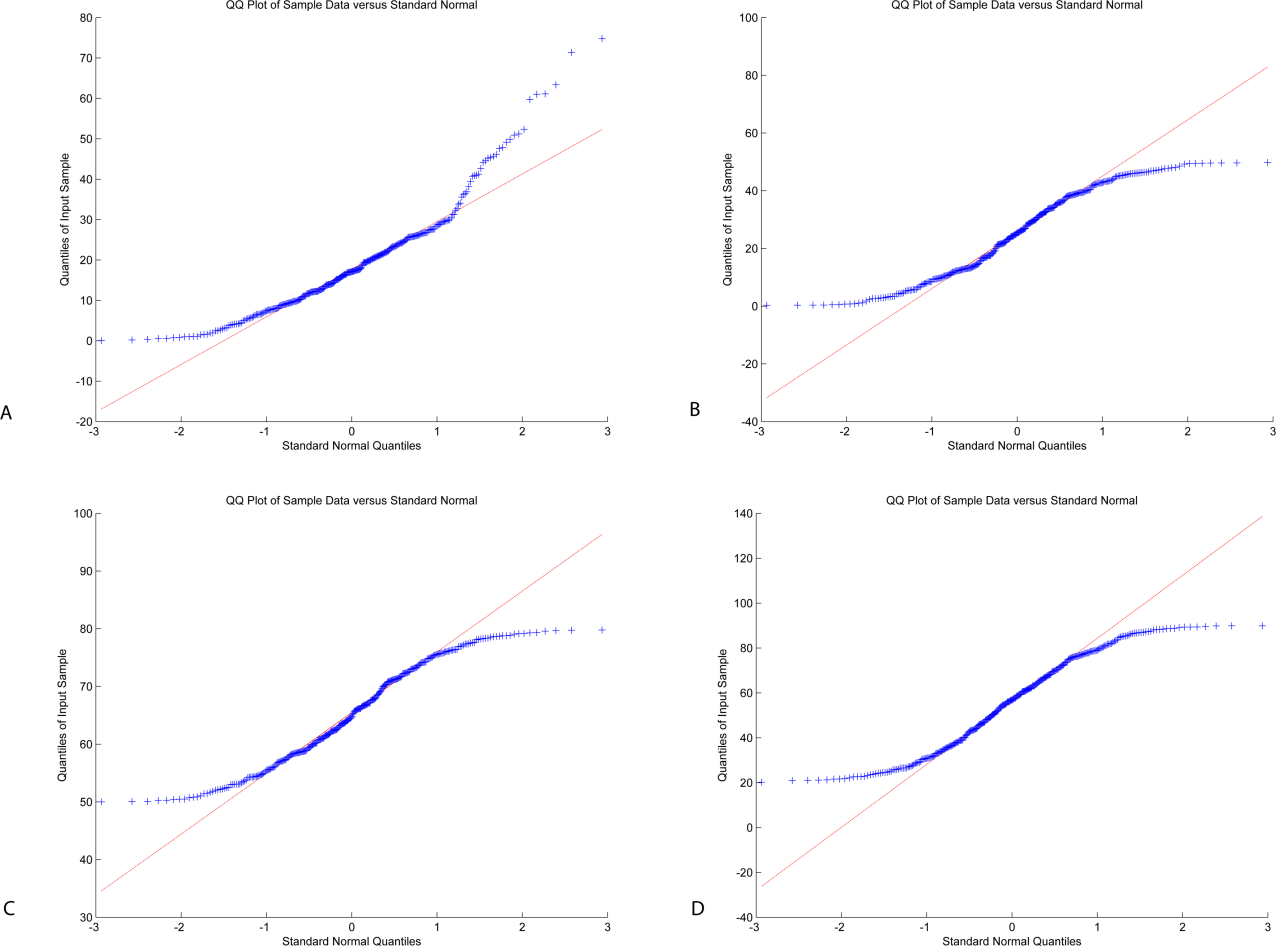
Q–Q plot: Neurosky (A, B) and Emotiv (C, D): attention (A, C) and meditation (B, D).

A Q–Q plot is a plot of the quantiles of two distributions against each other, which is used to compare the two distributions. Figure 7 illustrates the Q–Q plot for this sample suggesting that the data follow a nonlinear pattern, suggesting that the data are not distributed as a standard normal.

**Figure 8:**
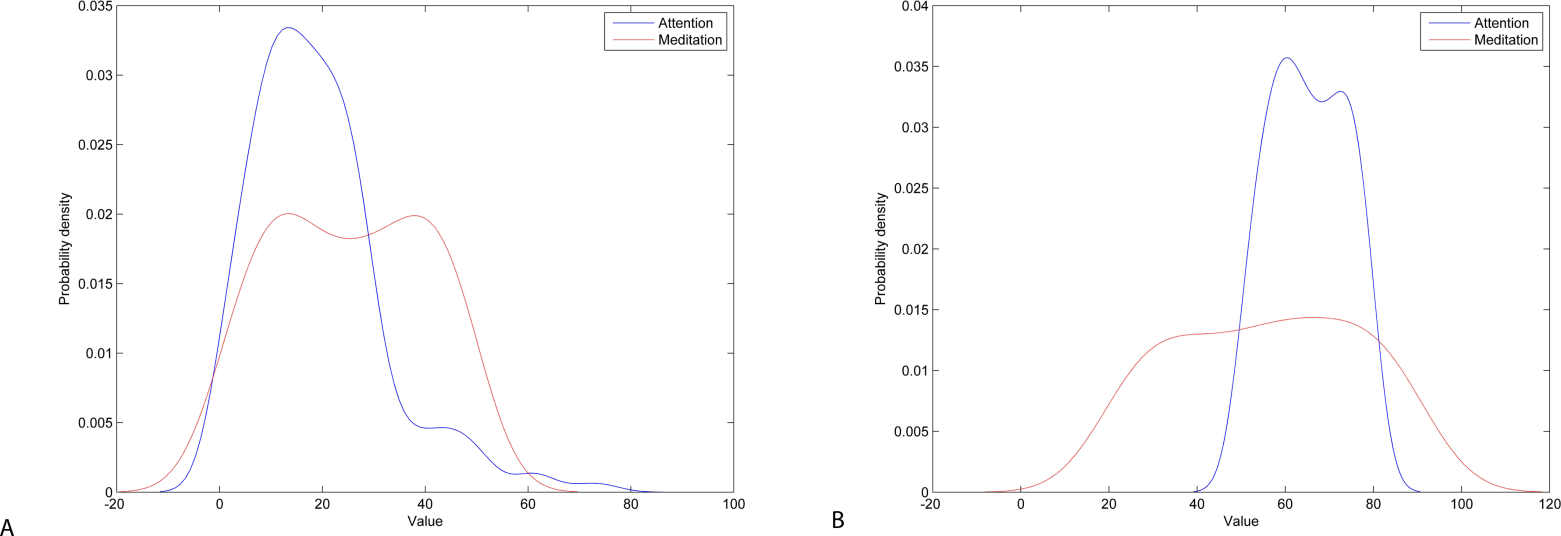
Probability density of attention and meditation data: Neurosky (A) and Emotiv (B).

The probability density function (PDF) plot of attention and meditation shows that both data overlap significantly for the Neurosky device making separation of user’s mental states very difficult (Fig. 8, left). However, for the Emotiv device data, the separation is larger making it easier to separate (Fig. 8, right). Furthermore, meditation may have a bi-modal PDF indicating that the signal may have more than one independent source.

As a criterion for estimating the separation between PDFs of attention and meditation, we use the Jaccard distance metric, which measures dissimilarity between sample sets. Here we use Jaccard to measure distance between overlapping and total areas under curve of PDF as follows:

(1) \begin{eqnarray*}J({f}_{1},{f}_{2})= \frac{\int {|}{f}_{1}(x)-{f}_{2}(x){|}dx}{\int {f}_{1}(x)dx+\int {f}_{2}(x)dx} ,\end{eqnarray*}

here, *f*_1_ and *f*_2_ are the PDFs of attention and meditation data, respectively.

The results presented in Fig. 8 show that the Jaccard distance between PDFs of attention and meditation data for the Neurosky data is 0.2890, while the distance for the Emotiv data is 0.4924 (using trapezoidal method for numerical integration), which means that the attention and meditation data is separable more easily in case of the Emotiv data.

**Figure 9:**
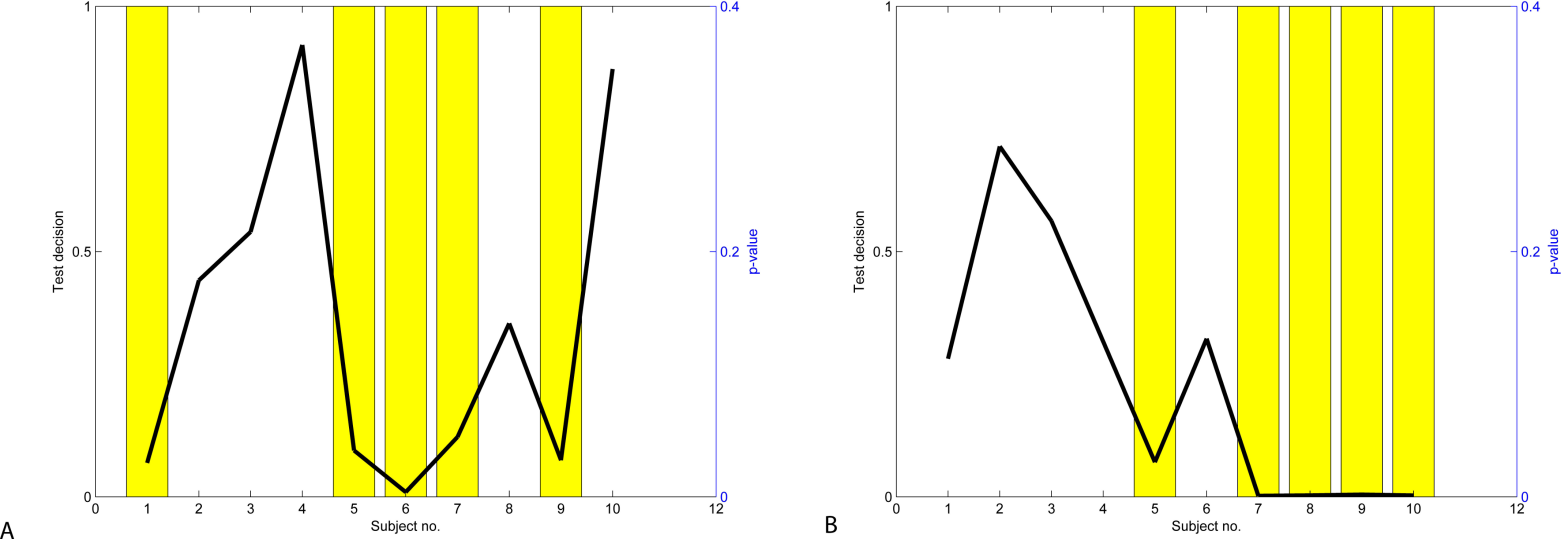
Results of *t*-test: Neurosky data (A) and Emotiv data (B).

To evaluate if attention and mediation data are significantly different from each other, we perform the Student’s two-sample unpaired *t*-test. The *t*-test performed on all data indicates that the hypothesis is rejected with *p* = 4⋅10^−8^ for the Neurosky data and *p* = 2.5⋅10^−12^ for the Emotiv data. The results of the *t*-test performed on the individual user data in presented in Fig. 9. The hypothesis is rejected for Subjects 1, 5, 6, 7 & 9 and confirmed for Subjects 2, 3, 4, 8 & 10 when using the Neurosky device. The hypothesis is rejected for Subjects 1, 2, 3, 4 & 6 and confirmed for Subjects 5, 7, 8, 9 & 10 when using the Emotiv device.

The relationship between attention and meditation values can be assessed using correlation. Table 2 presents the correlation results. The results show that there is a significant (weakly significant at *p* = 0.10) positive correlation (0.79) between attention and meditation levels of subjects for whom the hypothesis about difference of attention and meditation data has been confirmed.

**Figure 10:**
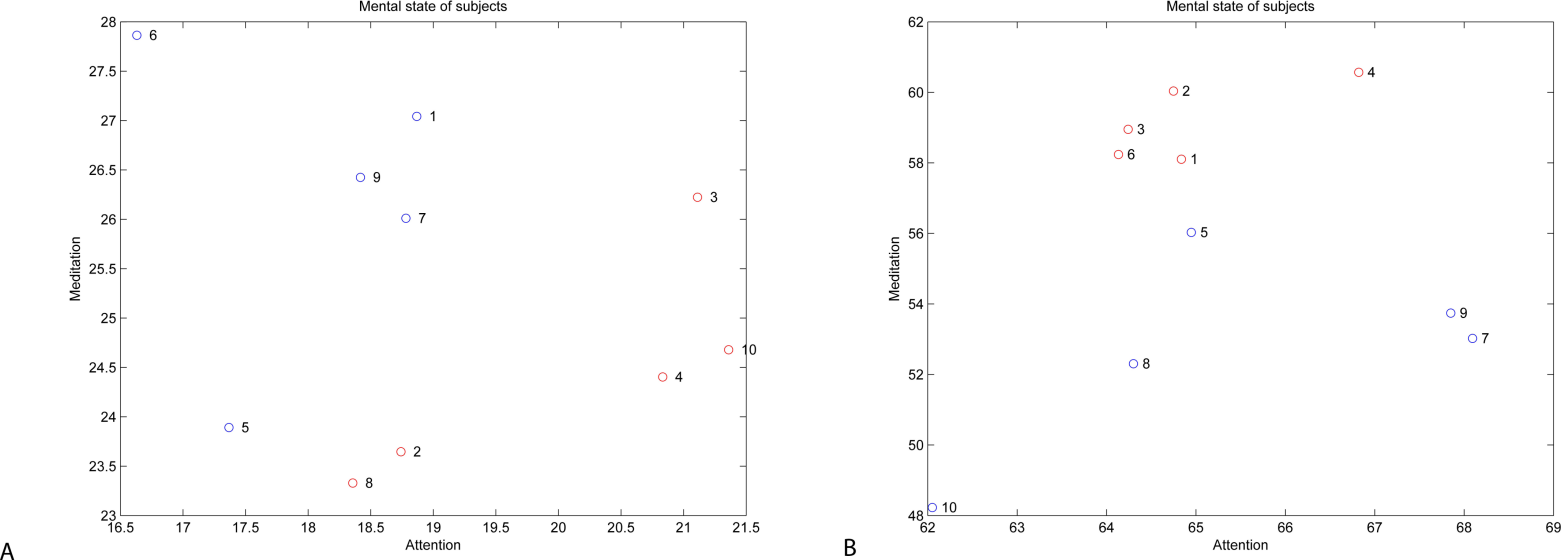
Levels of attention vs. meditation: Neurosky data (A) and Emotiv data (B).

When analysing the attention vs. meditation data (Fig. 10), we can clearly see that two groups of subjects identified using *t*-test and confirmed by correlation can be clearly identified in the attention vs. meditation plot. We claim that these two groups of subjects represent subjects, who were not able to master the BCI interface (in red) vs. subjects who have learned to use the BCI interface (in blue).

**Table 2:** Correlation between attention and meditation.

Device	Correlation between same-subject mean values	Correlation between same-subject mean values for subjects with rejected *t*-test hypothesis (*h* = 1)	Correlation between same-subject mean values for subjects with confirmed *t*-test hypothesis (*h* = 0)
Neurosky	−0.1932	−0.0245	0.79
	(*p* = 0.5927)	(*p* = 0.9688)	(*p* = 0.1072)
Emotiv EPOC	0.1913	0.5985	0.7340
	(*p* = 0.5966)	(*p* = 0.2863)	(*p* = 0.1579)

**Figure 11:**
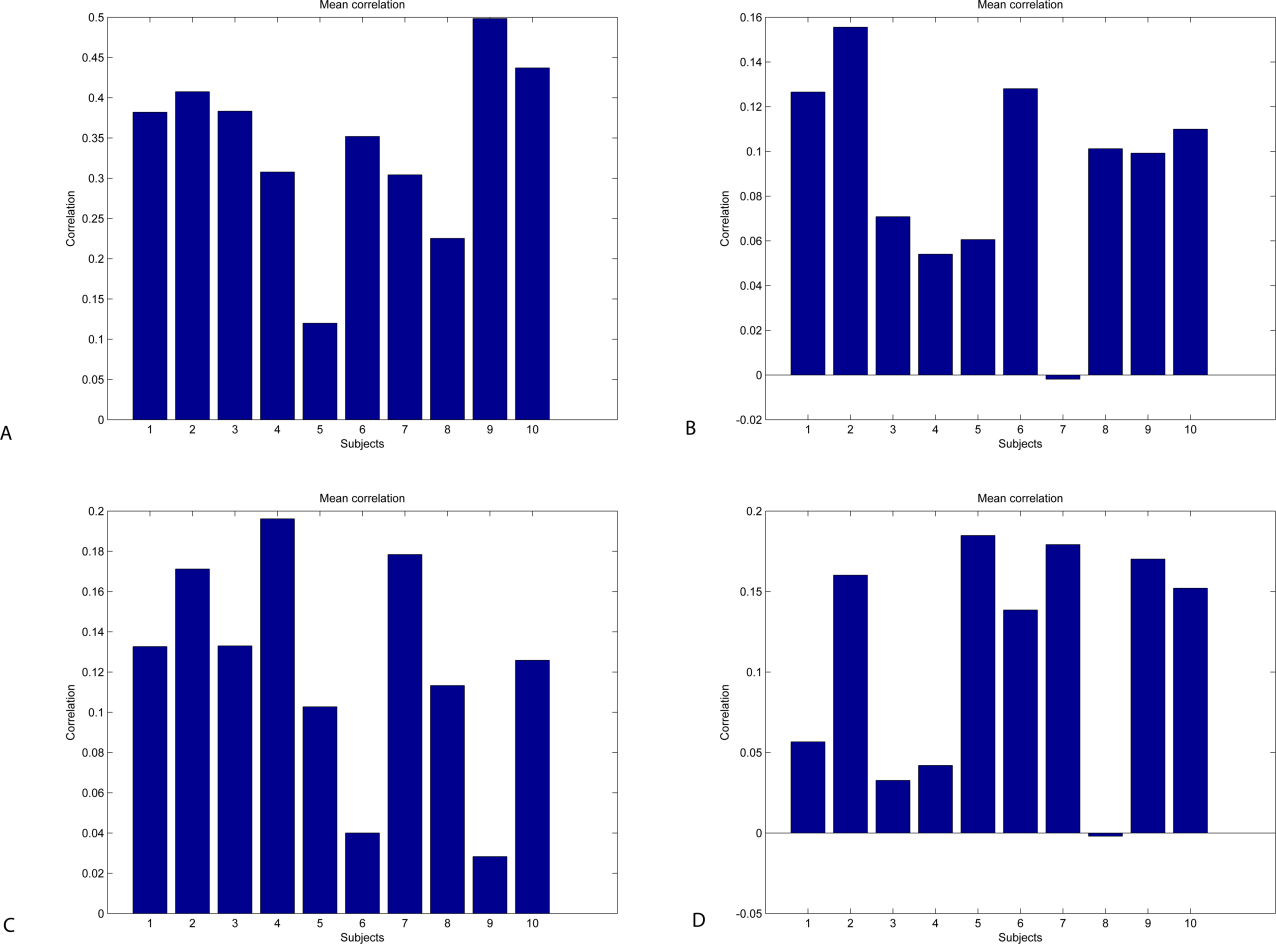
Inter-subject cross-correlation plot for Neurosky (A, B) and Emotiv (C, D): attention (A, C) and meditation (B, D).

An important measure is cross-correlation between mental states of different subjects. Given the same conditions and settings of the experiment, we assume that the results of subjects who have successfully mastered the BCI interface will have a more significant correlation than the results of BCI illicit users. In the case of the latter, the signal will be meaningless. The result of cross-correlation between subjects is presented in Fig. 11. Using the Neurosky device, cross-correlation for meditation is weak for all subjects, which means that all subjects were not able to achieve the relaxed state (the weakest for Subject 7 (*ρ* = 0.019)). For the attention state, the strongest absolute cross-correlation was achieved by subject 9 (*ρ* = 0.4984) and the weakest—for Subject 5 (*ρ* = 0.1749). Using the Emotiv device, cross-correlation for attention and meditation is weak for all subjects, which means that all subjects were not able to achieve the relaxed state.

**Figure 12:**
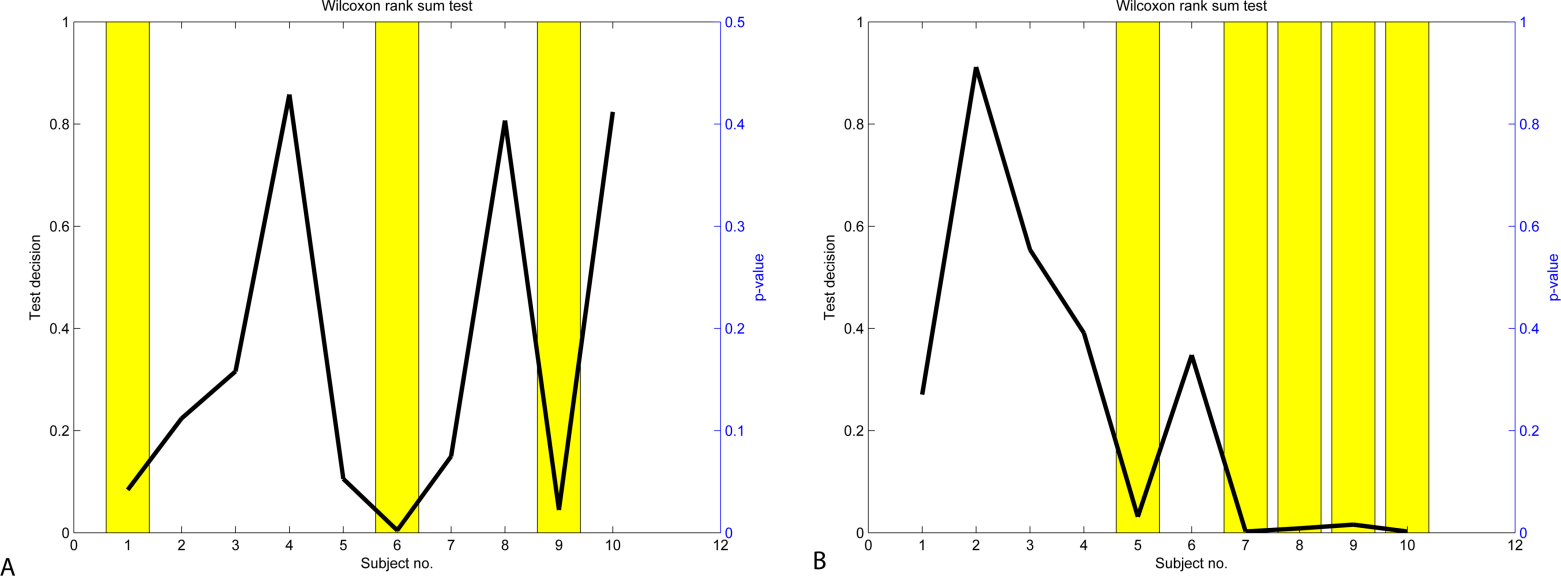
Results of Wilcoxon rank-sum test: Neurosky data (A) and Emotiv data (B).

In order to determine if difference between values of attention and meditation were statistically significant, we applied the Wilcoxon rank-sum test with the hypothesis that the medians of two variables differ. The results of the Wilcoxon rank-sum test performed on the individual user data in presented in Fig. 12. The hypothesis is rejected for Subjects 1, 6 & 9 and confirmed for Subjects 2, 3, 4, 5, 7, 8 & 10 when using the Neurosky data. The hypothesis is rejected for Subjects 5, 7, 8, 9 & 10 and confirmed for Subjects 1, 2, 3, 4 & 6 when using the Emotiv data. These results confirm the results of *t*-test (see Fig. 9) though the significance values differ.

**Figure 13:**
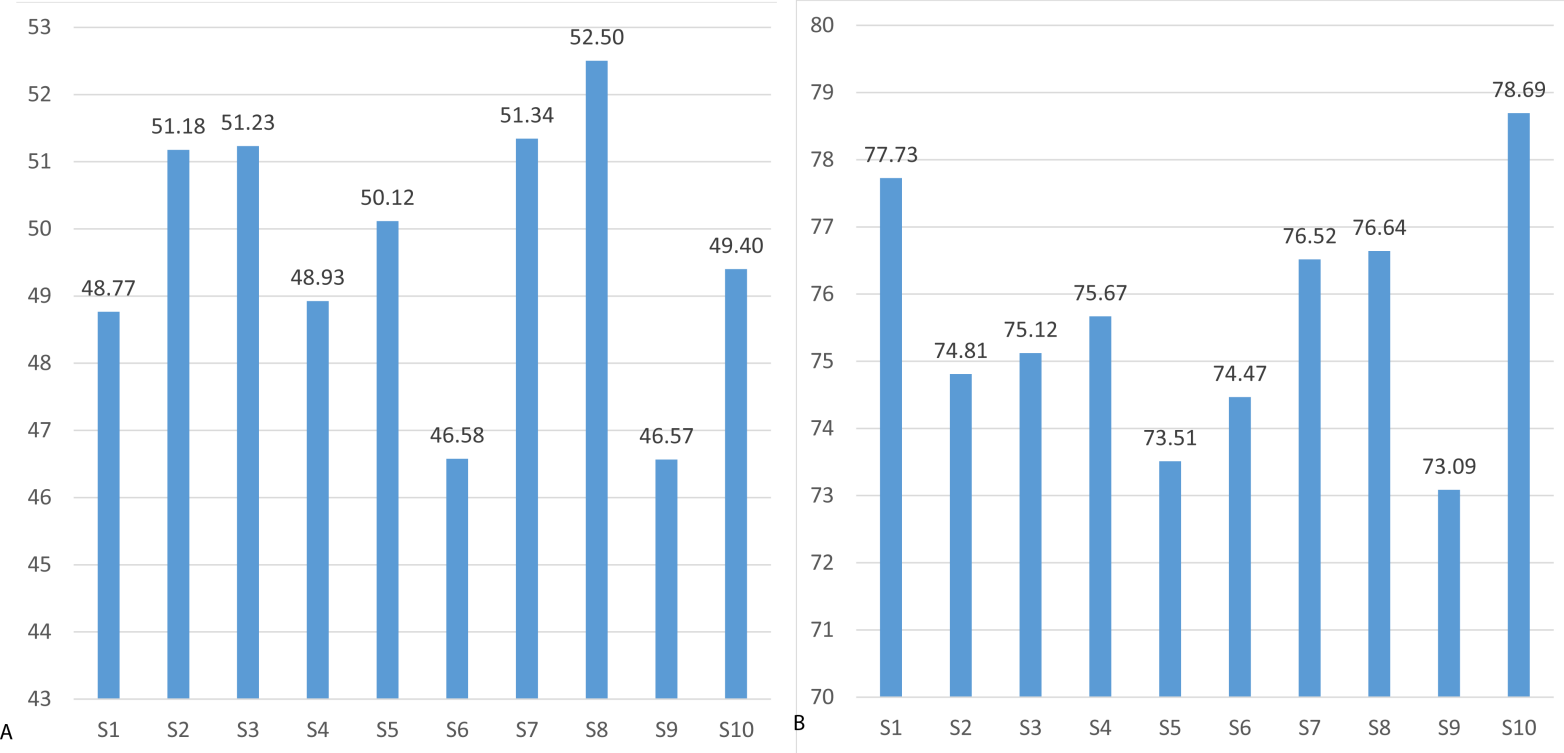
Mean accuracy of blinking recognition using Neurosky (A) and Emotiv EPOC (B).

**Table 3:** Descriptive statistics of blinking recognition results.

Metric	Min	Max	Mean	Std	Skewness
***Neurosky MindWave***					
Blinking	0.0213	99.8073	49.6616	28.7706	0.0143
***Emotiv EPOC***					
Blinking	50.1252	99.9896	75.6249	14.6053	−0.0359

**Figure 14:**
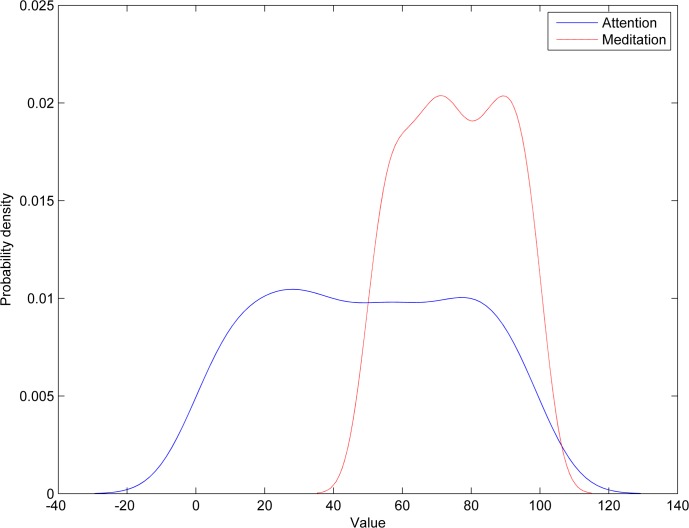
Probability density functions of Neurosky and Emotiv data in blinking recognition.

### Blinking recognition

The blinking recognition measurement results are presented in Fig. 13. The descriptive statistics of data are presented in Table 3. The results of Wilcoxon rank-sum test show that Emotiv EPOC is significantly better than Neurosky MindWave in recognizing eye blinking.

The results show that blinking recognition failed when using the Neurosky device with mean recognition accuracy of 49.6%, while the Emotiv device has achieved a satisfactorily recognition accuracy of 75.6%. The probability density functions of the Neurosky and Emotiv data (see Fig. 14) clearly illustrate the advantage of the Emotiv device in blinking recognition. Note the bi-modality of both data.

To test if the distribution of data is unimodal or bi-modal, we perform the Hardigand Dip Test. The Dip Test measures multimodality in a sample by the maximum difference, over all sample points, between the empirical distribution function, and the unimodal distribution function that minimizes that maximum difference. The Dip Test results show that the probability of the distribution is not unimodal.

Finally, we use the Friedman test to compare the data returned by both devices. The Friedman test checks if the measured average ranks are significantly different from the mean rank that is expected under the null-hypothesis. The results of the test *p* = 5⋅10^−37^ < 0.05 show that the performance of both devices as judged by their accuracy of recognizing blinking is significantly different.

## Discussion

Overall recognition accuracy of both the concentration (attention) and relaxation (meditation) mental states of subjects were 60.5% (for the Emotiv device) and only 22.2% (for the Neurosky device), which is not very usable in a practical sense. Attention was recognized more accurately than meditation, by 4–15% (mean = 9.3%) for the Emotiv device and by 3–11% (mean = 6.3%) for the Neurosky device. These results agree with the results achieved by other authors (e.g., see Liu, Chiang & Chu, 2013; Larsen, 2011). The reasons for that may be the similarity of features of attentive EEG data between subjects, while the meditation features are much more individual and are difficult to generalize. For the blinking recognition task, the Neurosky device achieved mean recognition accuracy of less than 50%, while the Emotiv device has achieved a satisfactorily recognition accuracy of more than 75%. We have also noticed that further into testing of different devices, the overall recognition accuracy increased by a few percent due to familiarization of subjects with both the devices and experiment conditions.

It is also very important to note that it is actually very hard for a person to produce a stable control signal (thus a command, by either focusing or relaxing). The process itself is very tiring and over excessive time has a significant input on recognition accuracy. Based on the above, we might argue that no matter what marketing material might tell you—a consumer EEG device is only suitable for a beginner level brain signal measurement and research and usable as a control device via its direct function—brain reading—only if a person has really no other mean of signal input (like in a complete locked-in paralysis state), otherwise other means of command recognition (be it gaze tracking, blinking, sound, video, etc. recognizers) are much more viable.

The results of measurement of blinking (signals) via a consumer EEG device are also not very promising. There is an obvious factor of external disturbances and signal itself is not very stable, especially when compared with devices built for this purpose and capabilities of sensors placing near the working field of eye muscles. The overall recognition accuracy ranges from around 50% for devices with low number of sensors (such as MindWave) to around 75% for devices with larger number of sensors (such as EPOC), overall in our opinion still too low for any practical control applications. On the other side, we noticed that a gaze tracking device was faster and more accurate than both EEG and EMG devices.

Other problems with the Emotiv EPOC are as follows: electrodes mounted on plastic springs are prone to shift along scalp. The EPOC also lacks EOG inputs normally used in eye artifact suppression. Furthermore, the impedance of saline solution electrodes is unstable due to drying. A common problem of both devices reported by subjects was external distractions such as the people standing around, which produced unwanted results from the lack of concentration, confirming the same observation also reported by other authors (e.g., see Vourvopoulos & Liarokapis, 2014).

Participants also noted discomfort after wearing the headset for an extended period of time. Therefore, we tried to limit the amount of time which participants wore the headset to reduce this discomfort. This discomfort is most likely due to the sideways pressure that holds the headset in place. Other studies also noted participants experienced discomfort (Haapalainen et al., 2010).

Given these limitations and the lack of conclusive findings, we would deem this device to be unsuitable for use in serious applications, which agrees with conclusions of other researchers (see, e.g., Harrison, 2013).

The problem of BCI illiteracy is especially acute with customer grade devices, as the low number of sensors, low quality of electrodes and inability to place the sensors accurately on the scalp reduces the quality of EEG data obtained thus hindering the establishment of effective feedback between the user and the system. Further studies are needed to better understand the underlying cause of BCI illiteracy and identify new BCI training procedures that will alleviate the BCI illiteracy.

Our findings, however, must be considered with the limitations of the study in mind: the number of subjects was small (only 10), there was no gender balance (nine males and only one female), all subjects were young people, all subjects were considered healthy, but there was no formal medical examination of their health condition done.

## Conclusions

We have performed usability testing of the Neurosky MindWave and Emotiv EPOC devices for tasks that require concentration and relaxation of subjects as well as for the blinking recognition task. The results showed that the Emotiv EPOC device has performed better (as measured by the recognition accuracy) for all tasks (∼9% better using attention/meditation data and ∼25% better for eye blinking recognition).

Using consumer-grade EEG devices, BCI illiteracy is a significant problem due to technical limitations of devices and weak feedback. Our research shows that up to 50% of users may be BCI-illicit when using low-cost EEG devices to perform control tasks based on mental state (attention and meditation) recognition. Thus, the results of experiments can be seriously affected, because if the user is not able to produce stable and distinct EEG patterns, then no machine learning method or classification algorithm would be able to recognize them. Improving BCI universality (that is, reducing BCI illiteracy) should be a top priority in BCI research focus on non-medical applications. Improved environment setup, training and subject instructions can make BCIs more universal, to some extent.

We suggest performing pre-screening of users by using statistical tests (Student’s paired *t*-test or Wilcoxon rank-sum test) to address the BCI illiteracy problem before performing validation of the BCI-based control methods. Very careful setting up of the experiment and proper motivation environment must be set up for EEG device usage, e.g., it is almost impossible to achieve the mental state of meditation if the surrounding environment is noisy.

High variability and non-normality of attention and meditation data imposes new challenges for developers who wish to use levels of attention and meditation as input to control or alter interfaces. The baseline levels of attention and meditation must be established for each user individually. For effective use, the combination of EEG readings with other input modalities should be established. Work for the future includes the combination of EEG readings with such as gaze, body posture and facial expressions should be considered. In future work, more research will be performed using different kind of experimental setting, e.g., the Stroop Colour-Word Interference Test (Stroop, 1935), a well-known psychological test of selective attention.

## Supplemental Information

10.7717/peerj.1746/supp-1 Data S1 Data Data of attention/meditation and blinking recognition using Neurosky Mindwave and Emotiv EPOC.

## Additional Information and Declarations

### Data Availability

The following information was supplied regarding data availability: The data is available as a supplemental dataset.
